# The Interplay between Gaze Following, Emotion Recognition, and Empathy across Adolescence; a Pubertal Dip in Performance?

**DOI:** 10.3389/fpsyg.2018.00127

**Published:** 2018-02-13

**Authors:** Rianne van Rooijen, Caroline M. M. Junge, Chantal Kemner

**Affiliations:** ^1^Department of Experimental Psychology, Helmholtz Institute, Utrecht University, Utrecht, Netherlands; ^2^Department of Developmental Psychology, Utrecht University, Utrecht, Netherlands; ^3^Brain Centre Rudolf Magnus, University Medical Center, Utrecht, Netherlands

**Keywords:** gaze cueing, emotion recognition, empathy, pubertal dip, sex differences

## Abstract

During puberty a dip in face recognition is often observed, possibly caused by heightened levels of gonadal hormones which in turn affects the re-organization of relevant cortical circuitry. In the current study we investigated whether a pubertal dip could be observed in three other abilities related to social information processing: gaze following, emotion recognition from the eyes, and empathizing abilities. Across these abilities we further explored whether these measurements revealed sex differences as another way to understand how gonadal hormones affect processing of social information. Results show that across adolescence, there are improvements in emotion recognition from the eyes and in empathizing abilities. These improvements did not show a dip, but are more plateau-like. The gaze cueing effect did not change over adolescence. We only observed sex differences in empathizing abilities, with girls showing higher scores than boys. Based on these results it appears that gonadal hormones are not exerting a unified influence on higher levels of social information processing. Further research should also explore changes in (visual) information processing around puberty onset to find a more fitted explanation for changes in social behavior across adolescence.

## Introduction

People's eyes are very informative in social interactions. First, eye gaze can direct someone's attention toward a gazed at location (e.g., Friesen and Kingstone, 1998). One demonstration that people are sensitive to gaze cueing comes from gaze cueing experiments. The first gaze cueing experiment was conducted by Friesen and Kingstone (1998) who showed that people are faster in detecting a target when a preceding face looked at the location where the target would appear (congruent condition) compared to a situation where the preceding face looked in the opposite direction (incongruent condition). This is called the gaze cueing effect. Second, eyes can express emotions as well as intentions and desires. The eye region is highly informative when deducting another's mental state (Frischen et al., 2007). The ability to recognize emotions from the eye region is often tested with the Read the Mind in the Eyes Task (RMET; Baron-Cohen et al., 2001). During this task participants see an image of the eye region of a person and are asked to choose one out of four options which word describes best how the person in the picture is feeling.

Closely related to gaze following and emotion recognition from the eyes is empathy. Empathy is “the drive to identify another person's emotions and thoughts, and to respond to these with an appropriate emotion” (Baron-Cohen, 2002). In other words, empathy enables one to give appropriate emotional responses. There is a clear connection between one's ability to empathize and this person's eye-gaze pattern. People with high empathizing abilities fixate more on the eye-region of the people they are looking at compared to people with lower empathizing abilities (Cowan et al., 2014). This heightened attention for the eye-region might in turn result in better performances on gaze following and emotion recognition from the eyes.

Surprisingly, the investigation of the development of and interplay between empathy and sensitivity to another person's eyes remains sparse, especially over the period of adolescence (for a review on the interplay between different sociocognitive processes, see Happé et al., 2017). It is across adolescence that changes in social behavior and environment become most pronounced. The current study therefore examines the interplay between gaze following, emotion recognition from the eyes and empathy in a large group of children ranging from 8 to 16 years.

Investigating a group from pre-adolescence to adolescence further allows us to examine the influence of gonadal hormones on different social processes. Gonadal hormones released during puberty play a role in re-organizing cortical circuitry, and causally influence the neural basis of face processing, and more general social information processes (Scherf et al., 2012). Although one might expect a linear improvement with age from infancy to adulthood on basic face and emotion recognition, previous studies found a “dip” in performance around the pubertal age: around mid-pubertal age (12–13 years old) children show worse performance on face and emotion recognition tasks compared to younger children and adults (e.g., Carey et al., 1980; Diamond et al., 1983; McGivern et al., 2002; Peters and Kemner, 2017). This pubertal dip in performance might be initiated by the heightened levels of gonadal hormones, which results in a shift from a caregiver bias toward a peer bias (Scherf et al., 2012; but for other theories see Chung and Thomson, 1995). Adolescents develop specific peer oriented behaviors which prepare them for adult social roles, such as developing peer friendships and exploring romantic relationships (Motta-Mena and Scherf, 2017). Indeed, in a face memory task adolescents show worse performance for the recognition of adult faces compared to prepubertal children and adults. However, they perform better than the other age groups for the recognition of faces which matched their own pubertal status specifically, which not necessarily matched their own age (Picci and Scherf, 2016). Thus, pubertal status, irrespective of age, seems to influence the development of face processing abilities (see also Diamond et al., 1983; Lawrence et al., 2015).

If gonadal hormones indeed influence task performance by re-organizing cortical circuitry, processes dependent on similar underlying brain structures should all be affected by this hormonal influx. Evidence from studies using functional Magnetic Resonance Imaging (fMRI) suggest that the same brain areas involved in face-processing are also part of the brain circuitry involved in other social information processes such as the ones under investigation in the current study; gaze following, emotion recognition from the eyes and empathy. The core network involved in face-processing is centered around the superior temporal sulcus (STS), the fusiform face area and the occipital face area (Gobbini and Haxby, 2007). Likewise, studies on eye gaze processing highlight the involvement of the STS, together with the middle temporal gyrus and inferior parietal lobule, both in adults (e.g., Hoffman and Haxby, 2000) as well as in children (Mosconi et al., 2005; Vaidya et al., 2011). Similarly, fMRI studies looking into the RMET also show activation in the STS, as well as the temporal pole and the inferior frontal gyrus (IFG) (e.g., Adams et al., 2010; Castelli et al., 2010; Gunther Moor et al., 2012). For young adolescents (10–12 year-olds), the medial prefrontal cortex is involved as well (Gunther Moor et al., 2012). Finally, brain-imaging studies on empathy also observe activation in the STS region (e.g., Zaki et al., 2009; Dziobek et al., 2011), as well as a thinner cortex in this area in people with lower empathy skills (Hadjikhani et al., 2005). Taking all these studies together we can conclude that brain circuitries centered around the STS are crucial in social information processes such as gaze following, emotion recognition from the eyes as well as empathy.

Coupling the findings of great overlap in brain circuitry recruited for basic face recognition and for higher levels of social processing, and the behavioral observation that there is a dip in basic face recognition in puberty, we expect to find a pubertal dip in measures that tap into other social information processes as well. We focus here on three examples of higher social information processes; gaze following, emotion recognition from the eyes and empathy. Although, as far as we know, there are no studies that examine the interplay between all three of these processes across adolescence, there are a few studies that focus on the influence of gonadal hormones on one of our measures. For instance, there is one study that reports a dip in RMET performance around pubertal age (Vetter et al., 2013). Although this study found no reason to attribute the dip to pubertal status, note that the different pubertal groups were rather small and that the study sampled only children from 12 to 15 year old. Another study reports that for empathizing abilities there is a plateau rather than a puberty dip, with no increase in abilities between 10 and 14 years of age (Garaigordobil, 2009). To our knowledge no study examined the developmental changes over adolescence in performance on the gaze cueing task. With our study we will get a broader view on the effect of pubertal status on these social information processes.

To be able to take the pubertal status of the participants into account we included the Pubertal Development Scale (PDS), which is a written questionnaire that assesses multiple aspects of pubertal development (Petersen et al., 1988). Empathizing abilities will be measured with the Interpersonal Reactivity Index (IRI; Davis, 1980), which is a written questionnaire looking into several aspects of empathizing behavior. The IRI is found to be a reliable measure of empathy as it shows correlations with several other empathy measures (Riggio et al., 1989).

To look even more closely at the influence of gonadal hormones we will also examine sex differences. Sex differences partly reflect, among other influences such as genes and environment, the effects of gonadal hormones on the abilities under investigation in the current study. Differences in testosterone exposure might have a causal role in sexual dimorphism in social development (Chapman et al., 2006; Knickmeyer and Baron-Cohen, 2006). During adolescence there is a tremendous increase in testosterone in boys, yet not in girls (Schulz and Sisk, 2006), which might result in the initiation or enlargement of already existing sex differences. Studying gaze following, emotion recognition and empathy in an adolescent sample allows us to further investigate the developmental time course of the sex differences found in adults, with females consistently outperforming males (e.g., Bayliss et al., 2005; Deaner et al., 2007; Alwall et al., 2010; Kirkland et al., 2013; Baron-Cohen et al., 2015).

In sum, our aim with the current study is to test whether a pubertal dip, caused by heightened levels of gonadal hormones which in turn affects re-organization of cortical circuitry, can be observed in higher social information processing measures such as the gaze cueing task, the RMET, and the IRI. We expect to find this dip to be present across our measures as all processes rely on overlapping neural regions, which are similar to the regions involved in face recognition processes in which a pubertal dip is observed. Because of this similar underlying activation pattern we also expect that the performances on all three tasks will correlate with each other. Furthermore, we explore the influence of gonadal hormones more closely by looking at sex differences. We expect females to outperform males on all tasks.

## Materials and methods

### Subjects

A sample of 124 adolescents participated in this study (57 boys; mean age 12.0 years, *SD* = 2.61, range 8–16). The participants were recruited through advertisements at primary and high schools in and around Utrecht, the Netherlands. This study was embedded in the first round of a larger cohort study on the development of cognition at Utrecht University, the Consortium on Individual Development (https://www.uu.nl/en/research/dynamics-of-youth/youth). The project was approved by the Medical Ethical Committee of the University Medical Center of Utrecht. Participants and their parent(s)/caregiver(s) gave informed consent at the start of the study and received 10 Euro for the test session.

### Stimuli

#### Gaze cueing task

Stimuli consisted of faces with a neutral expression of 10 different identities, 5 male and 5 female, taken from the MacBrain Face Stimulus Set. Of each identity, three different pictures were used. One with a direct gaze, and two with an averted gaze to either the left or the right. Pictures were in grayscale and had an oval cutoff such that hair and background were not visible. The pictures had a width of 738 pixels and a height of 981 pixels, with the eyes at a height of 440 pixels. The target picture could either be a bird, cow, pink flower, red flower, or spiral (size 150 × 150 pixels).

#### Reading the mind in the eyes task

We used an adapted version of the RMET which was translated to Dutch and suitable for use with children and adolescents (Overgaauw et al., 2014). Stimuli consisted of 28 pictures of the eye region of a face, expressing a certain feeling or emotion (size 541 × 214 pixels). Each stimulus had a different identity. The pictures were accompanied by 4 words that describe possible feelings and emotions. One of these words was the target word describing the mental state of the individual in the picture.

### Questionnaires

#### Interpersonal reactivity index

We used the Interpersonal Reactivity Index (IRI; Davis, 1980) as a measure of empathizing abilities. In the current study we used a Dutch version which was adapted for use with children and adolescents (Hawk et al., 2013). The IRI consists of four scales of seven items each, which had to be answered on a five-point scale ranging from 0 (doesn't describe me well at all) to 4 (describes me very well). We looked at three of the four subscales of this questionnaire (“Perspective Taking,” “Fantasy,” and “Empathetic Concern”), as these subscales are related to “sensitivity to others” (Davis, 1983), important in the tasks of the current study. We computed a score for every of the three subscales by adding the scores for all the 7 items belonging to that subscale. Then, a total score was computed by adding the scores of the three subscales. One participant did not complete this questionnaire.

#### Pubertal development scale

To measure the pubertal status of the participants we used the Pubertal Development Scale (PDS; Petersen et al., 1988), translated in Dutch. This questionnaire assesses multiple aspects of pubertal development, focused on physical changes of the body. For each sex there are five questions which can be answered on a 4-point scale ranging from 1 (maturation not started) to 4 (maturation completed). An overall pubertal development score was computed by averaging across the five items. Based on the answers children can be classified into one of five categories; prepubertal (1), early pubertal (2), midpubertal (3), late pubertal (4), or postpubertal (5). To compare our results with previous research (Vetter et al., 2013) we reclassified the children into three groups; prepubertal (group 1 & 2; 42 boys, 24 girls), midpubertal (group 3; 13 boys, 11 girls), and postpubertal (group 4 & 5; 4 boys, 27 girls)^1^. Three participants did not complete this questionnaire.

### Procedure

The participants came to our research facility for an entire testing day, consisting of several tasks to measure different aspects of cognition. The tasks described in the current paper were two of them. The questionnaires were completed during this same testing day. Due to randomization the participants completed the two tasks at different moments during the day and in a counterbalanced order. Both tasks were programmed in Matlab version R2013a (MathWorks Inc., USA) and the Psych-Toolbox (version 3.0.11, Brainard and Vision, 1997) running on a MacBook Pro (OS X 10.9).

#### Gaze cueing task

The gaze cueing task was conducted with a Tobii TX 300 eye-tracker (sample rate 300 Hz) integrated with a computer screen (1920 × 1080 pixels; size 23 inch; refresh rate 60 Hz). Participants were seated at 65 cm distance from the screen and a chin-rest was used to stabilize head position. First, the task was explained and participants were instructed to look at the face and then look at the target as soon as it appeared on the screen. A 5-point calibration was performed and after accepted calibration or re-calibration the task started. The task consisted of 80 trials in total, 20 trials for each condition (left and right congruent/incongruent), in random order. Each face identity was shown twice for each condition. A trial started with a bouncing fixation dot in the middle of the screen (50 × 50 pixels).

The trial continued once the participant had focused on the fixation dot for a period of 36 samples. Then, a face with direct gaze was presented for 300 ms, followed by a face with an averted gaze for a random duration between 300 and 500 ms (over all trials the average duration was 400 ms). Next, a target was shown at either the right or left side of the screen, and started to spin when the participant fixated on it (i.e., three eye samples in an area of 200 pixels around the target), or after an elapsed time of 1500 ms in which the participant had not fixated on the target. The target remained spinning for 1000 ms. The task was automatically paused after 40 trials, the participants could indicate themselves when to continue.

#### Reading the mind in the eyes task

The Reading the Mind in the Eyes task consisted of 28 trials in total, preceded by one practice trial. In each trial, a picture of an eye region appeared on the screen, accompanied by four words, each describing a feeling or emotion. The participant was instructed to select the word which described the mental state of the person in the picture best. There was no time limit for answering. At the end of the task the participant received feedback on how many answers were correct. We scored on how many trials the participant had correctly interpreted the emotion in the eyes. One participant did not perform this task, so the results are based on a total sample of 123 participants.

#### Data reduction of the gaze cueing task

Fixations were determined with the Identification by 2-Means Clustering algorithm (I-2MC; Hessels et al., 2016). This algorithm is able to detect fixations in data with possibly high noise levels, both within and between participants and trials. Therefore, it is specifically useful for infant and child eye-tracking data. In the present study, periods of data loss up to 100 ms in the raw data were interpolated using Steffen interpolation if at least two samples of valid data were available at each end. For fixation detection we used a moving window of 200 ms width. Fixations that were not more than 30 pixels apart and that were separated by no more than 30 ms were merged. Fixations with a total duration shorter than 40 ms were removed.

In the analysis, we looked at target-driven and anticipatory saccades. A saccade was defined as (I) a fixation during target presentation on the target position and (II) the preceding fixation was on the face-stimulus until either target onset (i.e., target-driven saccade) or until at least 80 ms after cue-onset (i.e., anticipatory saccade). Target-driven saccades occurred in 40.5% of the trials, and 26.1% of the trials were anticipatory saccades. Participants were excluded from analysis when there were less than 10 included trials in one or more conditions (*n* = 6), eventually resulting in a total sample of 118 participants. For each participant the median latencies of the saccades per condition were calculated, defined as the time between target-onset and the start of the first fixation on target location. In addition, we calculated a difference score (RT on incongruent trials—RT on congruent trials) to examine the gaze cueing effect.

## Results

### Pubertal status effects

#### Gaze cueing task

**Figure 1:**
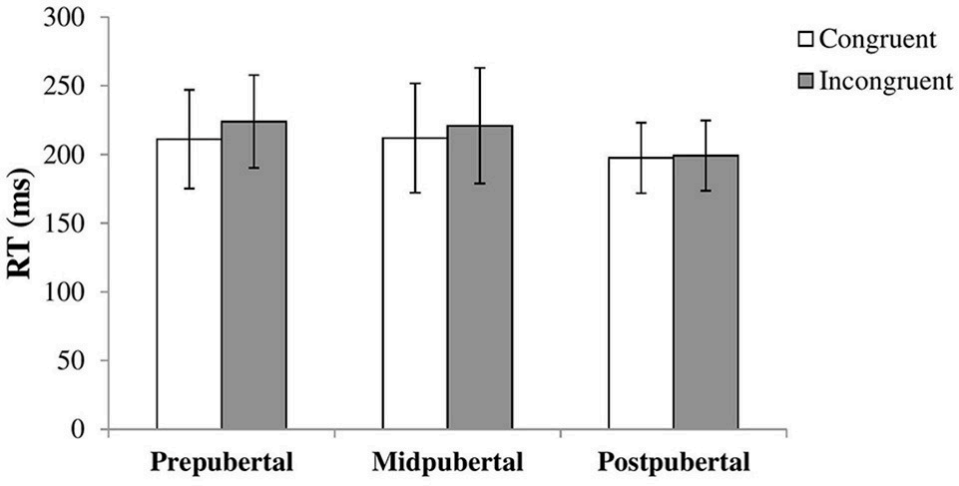
Bars shown represent reaction times in ms, separate for congruent and incongruent trials, per pubertal group. Error bars show the standard deviation from the mean. Data showed a main effect for congruency with higher RTs for incongruent trials, and a main effect for pubertal status with lower overall RTs for the postpubertal group. There was no significant interaction effect.

We performed a repeated measures ANOVA with congruency as within-subjects factor and pubertal status as between-subjects factor. A main effect for congruency [*F*_(1, 113)_ = 14.50, *p* < 0.001, η2 = 0.11] showed that the RTs for congruent trials (M = 207.8, *SD* = 34.66) were faster than RTs for incongruent trials (M = 217.0, *SD* = 35.14). There was a main effect for pubertal status as well [*F*_(2, 113)_ = 3.77, *p* = 0.026, η2 = 0.06]. *Post-hoc* tests showed that the postpubertal group showed faster overall RTs compared to the prepubertal [*t*_(73.56)_ = 3.12, *p* = 0.003, Cohen's *d* = 0.64, equal variances not assumed] and midpubertal [*t*_(52)_ = 2.05, *p* = 0.046, Cohen's *d* = 0.57] group. There was only a marginally significant interaction effect between congruency and pubertal status [*F*_(2, 113)_ = 2.98, *p* = 0.055, η2 = 0.05]. Figure 1 shows the results per pubertal group. Inspecting the graph suggests that the gaze cueing effect declines with pubertal status, but we did not further examine this as the interaction was not significant.

#### Reading the mind in the eyes task

**Figure 2:**
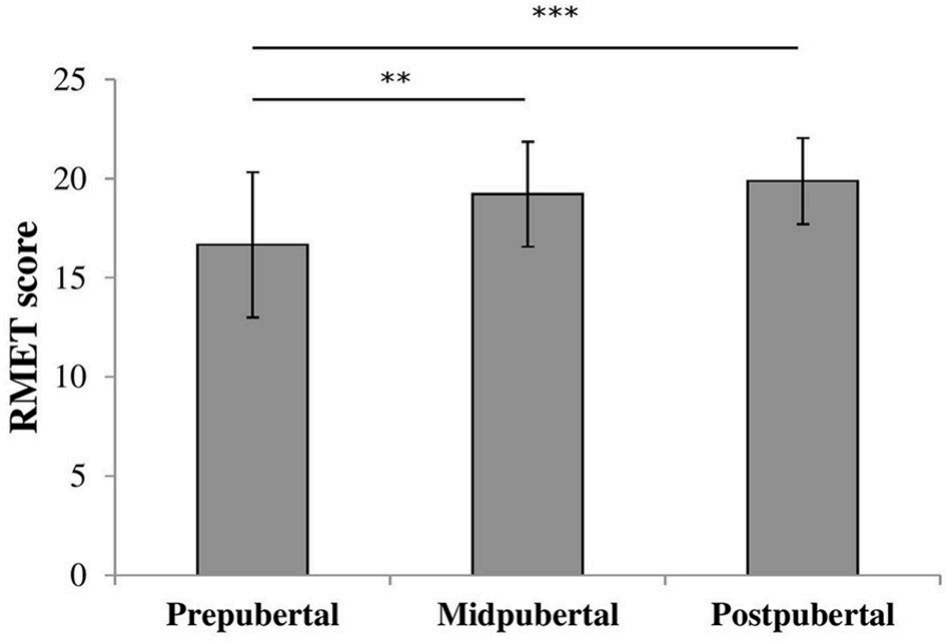
Bars shown represent total RMET score per pubertal group, error bars show the standard deviation from the mean. Data showed a main effect for pubertal group. ^**^p < 0.005; ^***^p < 0.001.

To check for the effect of pubertal status we performed an oneway ANOVA on RMET scores with pubertal status group as between-subjects factor. There was a significant effect for pubertal status [*F*_(2, 117)_ = 10.37, *p* < 0.001, η2 = 0.15], shown in Figure 2. *Post-hoc* analyses revealed that prepubertal children (M = 16.8, *SD* = 3.62) scored significantly lower compared to both midpubertal [M = 19.2, *SD* = 2.65; *t*_(87)_ = −2.95, *p* < 0.005, Cohen's *d* = 0.71] and postpubertal children [M = 19.7, *SD* = 2.47; *t*_(82.43)_ = −4.48, *p* < 0.001, Cohen's *d* = 0.87, equal variances not assumed]. The scores of the midpubertal and postpubertal children did not differ significantly [*t*_(53)_ = −0.63, ns].

#### Empathy

**Figure 3:**
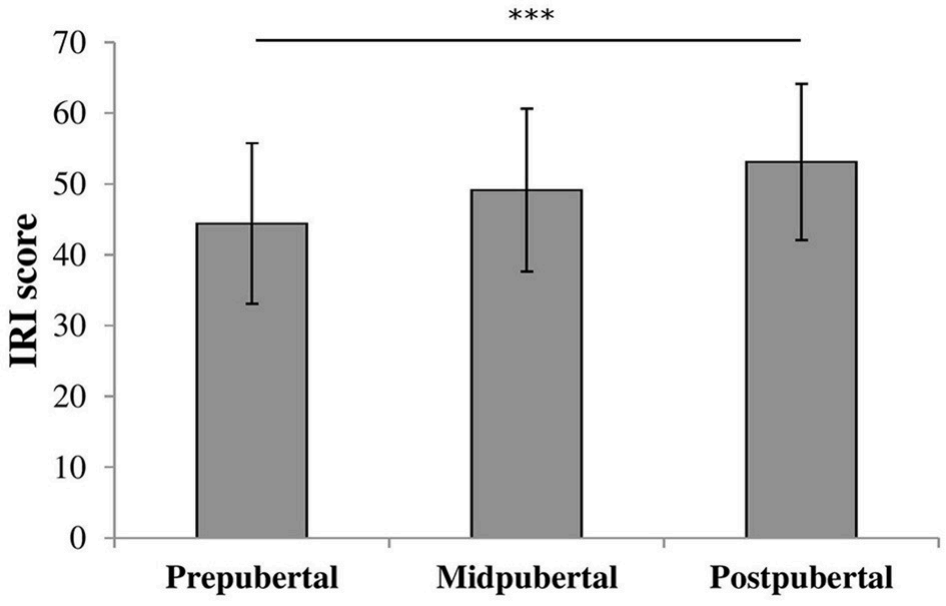
Bars shown represent total IRI score per pubertal group, error bars show the standard deviation from the mean. Data showed a main effect for pubertal group. ^***^p = 0.001.

We performed an oneway ANOVA on IRI scores to examine differences in empathizing abilities between the pubertal groups. There was a significant effect for pubertal status [*F*_(2, 117)_ = 6.47, *p* = 0.002, η2 = 0.10], shown in Figure 3. *Post-hoc* analyses showed that prepubertal children (M = 44.3, *SD* = 11.25) scored similar to midpubertal children [M = 49.1, *SD* = 11.51; *t*_(87)_ = −1.799, ns] and significantly lower than postpubertal children [M = 52.8, *SD* = 10.93; *t*_(94)_ = −3.525, *p* = 0.001, Cohen's *d* = 0.78]. Midpubertal and postpubertal children did not differ in IRI scores [*t*_(53)_ = −1.221, ns].

### Sex differences

#### Gaze cueing task

To check for sex differences on the gaze cueing task we performed a repeated measures ANOVA with congruency (congruent vs. incongruent) as within-subjects factor and gender as between-subjects factor. Results showed a significant main effect for congruency [*F*_(1, 116)_ = 23.24, *p* < 0.001, η2 = 0.17], where RTs for congruent trials (M = 208.1, *SD* = 34.62) were faster than RTs for incongruent trials (M = 217.1, *SD* = 34.95). There was no significant main effect for gender [*F*_(1, 116)_ = 0.00, ns], nor an interaction effect [*F*_(1, 116)_ = 2.13, ns].

#### Reading the mind in the eyes task

We performed an independent samples t-test to check for gender differences on the RMET score. There was no significant difference in scores between boys (M = 17.5, *SD* = 3.43) and girls [M = 18.4, *SD* = 3.43; *t*_(121)_ = −1.504, ns].

#### Empathy

To examine whether boys and girls differed in their empathizing abilities, we performed an independent samples t-test on the IRI score. Boys (M = 42.5, *SD* = 11.04) scored significantly lower on the IRI compared to girls [M = 52.0, *SD* = 10.37; *t*_(121)_ = −4.942, *p* < 0.001, Cohen's *d* = 0.90].

### Correlations between gaze cueing, the RMET, and empathy

We examined whether the three abilities tested in the current study are related, as we expected based on similar underlying brain activation and previously reported correlations between empathy measures and both gaze cueing and the RMET (Baron-Cohen et al., 2001; Bayliss et al., 2005).

As both the gaze cueing difference score and the RMET score violated the assumption of normality, as indicated by significant Kolmogorov-Smirnov values [difference score: D(118) = 0.085, *p* = 0.035; RMET score: D(118) = 0.114, *p* = 0.001], Spearman correlations were conducted. There was a significant negative correlation between the gaze cueing difference score and the RMET score (r_s_ = −0.223, *p* = 0.015). This means that individuals who are highly influenced by the cue validity (big difference in RT between congruent and incongruent trials) are worse in emotion recognition from the eyes compared to individuals who are less influenced by the cue validity.

There was no significant correlation between the IRI score and the gaze cueing difference score (r_s_ = −0.058, ns). Thus, gaze following does not seem to be related to empathizing abilities. We found a significant positive correlation between the IRI score and the RMET score (r_s_ = 0.364, *p* < 0.001). This means that individuals who were good in emotion recognition from the eyes also show higher empathizing abilities.

## Discussion

During puberty a dip in face recognition is observed (Diamond et al., 1983; Picci and Scherf, 2016), possibly caused by heightened levels of gonadal hormones which in turn affects re-organization of cortical circuitry. In the current study we investigated whether a pubertal dip could be observed in three other abilities related to social information processing; gaze following, emotion recognition from the eyes and empathizing abilities. All of these three abilities rely on brain circuitries centered around the STS, as does face recognition, and therefore we expected that these abilities are influenced by the heightened hormone levels in puberty in similar ways. For the same reasoning we also expected that performances on all three measurements would be correlated. We further explored the effects of gonadal hormones on these tasks by examining sex differences. In the following sections we will first discuss pubertal status effects and sex differences for each of the three measurements separately, after which we turn to the observed correlations across tasks. Last, we describe possible alternative explanations for the observed pubertal effects.

In our first task, the gaze cueing task, we observed an overall gaze cueing effect, with higher reaction times for incongruent compared to congruent trials. We observed no interaction effect with pubertal status nor a sex difference. Pubertal status influenced the overall reaction times, but not the magnitude in which the participants' attention was directed by the gaze cue. To our knowledge, this is the first study which looked at the development of the gaze cueing effect over adolescence. Our results show no development of the effect over this period, although a trend indicates a slight decline in the gaze cueing effect across adolescence. These results indicate that the gaze cueing effect is fairly robust and not easily influenced by individual factors. Previous studies reported gender differences in an adult population (Bayliss et al., 2005; Deaner et al., 2007), but we did not replicate this in the current sample of adolescents. Not many gaze cueing studies report on either the presence or absence of sex differences in their results. It is therefore hard to conclude whether sex differences arise after adolescence, whether sex differences arise during childhood but we failed to find them in the current sample or whether differences do not exist.

In our second task, the RMET, we observed a pubertal status effect, yet no sex difference was observed. The prepubertal children performed worse on this task compared to the mid- and postpubertal children, who did not differ in their performance. This result suggests that RMET performance first increases but later reaches a plateau. Whether there is more improvement on a later age cannot be determined based on the current study. Our results are in contrast with the study by Vetter et al. (2013) who did not find a pubertal status effect. Possibly our results are more representative as the sample size of the current study was twice as big compared to the study of Vetter et al. However, these are the only two studies looking at the influence of pubertal status on RMET performance. Further research is needed to confirm our results.

The third measurement, the IRI questionnaire, revealed both a pubertal status effect and a sex difference. Prepubertal children showed lower empathetic abilities than postpubertal children and girls had higher scores than boys on this questionnaire. These results suggest that there is a small gradual improvement in empathizing abilities over adolescence, only reaching significance when comparing the scores of the two outer groups. This is consistent with the finding of no increase in empathizing abilities between 10 and 14 years of age, which are children who are probably in the pre- or midpubertal phase (Garaigordobil, 2009).

Our next question was whether there are correspondences between the three different tasks. Recall that other studies observed a positive link between empathy and a person's eye-gaze pattern: High empathizing abilities are related to more fixations on the eye-region (Cowan et al., 2014). We therefore expected that empathy scores in our sample would positively correlate with the gaze cueing effect and RMET scores. Indeed, RMET scores in our study are positively related to empathizing abilities. However, we did not observe a correlation between empathy scores and the gaze cueing effect. Higher empathy does not influence a person's attentive behavior in response to gaze cues. Third, although we observed a correlation between the gaze cueing effect and RMET scores, it turned out to be negative. Apparently, individuals who perform well in emotion recognition from the eyes, are also the individuals who are less influenced by cue validity. We expected to find a positive correlation between these two measures, as they both rely on similar brain areas and tap into similar processes. We have no direct explanation for the negative correlation observed in our data. Further research should tap deeper into both processes to find differences which may explain our observed negative correlation. Indeed, while there is reason to believe that brain circuitry involved in these processes overlap to some extent, there is also evidence highlighting that gaze following and emotion recognition are distinct abilities, each additionally recruiting different areas in the brain. A study with women with Turner's Syndrome (lack of a complete X chromosome) shows that these women are impaired in emotion recognition from the eyes, yet not in gaze following, possibly due to dysfunction of the amygdala (Lawrence et al., 2003). This suggests that these two processes are dissociable and that at least the affective aspect of emotion recognition is supported by a distinct brain circuitry.

When looking at the correspondences across the three measurements, especially empathy and emotion recognition from the eyes seem to be related to each other at a correlational level. The relation with gaze following remains more unclear. Also the individual characteristics such as pubertal and sex effects that could possibly bear on these measurements do not all pattern alike. For example, our results show that pubertal status effects and sex differences do not consistently co-occur and do not show the same pattern for all tasks. Based on these results it is hard to pinpoint the exact effect of gonadal hormones on higher levels of social information processing. Clearly, this study shows that different sorts of higher social processing do not reveal similar levels of involvement of gonadal hormones. There are several possibilities why this is the case. One explanation could be that the lack of consistent puberty effects across different forms of social information processing highlights that gonadal hormones play only a minor role in higher levels of social information. Another explanation could be that gonadal hormones differentially modulate processing of social information, depending on the exact configuration of the task and ability at hand. It is also possible that the way in which we could easily quantify pubertal status (i.e., via self-based questionnaires) is more prone to subjective measurement error compared to direct measures of gonadal hormones. Research with more direct measures of gonadal hormone levels and brain activation would allow us to draw firmer conclusions about the role of these hormones in social information processes.

There are some other theories which might explain our observed pubertal effects. Diamond et al. (1983), and Soppe (1986) argued that instead of a direct effect of gonadal hormones there is a more indirect effect of pubertal changes on face encoding. Once children become aware of their own pubertal development they may subconsciously change their interests, for example changes in which aspects of a face are in the center of attention. For example, adults and young children show a left visual field advantage for unfamiliar faces, yet this advantage was not present in 12- and 14-year-olds (Diamond et al., 1983). These differences in attentive processes might cause a period of less efficient face processing. Basic processes of joint attention, such as eye gaze following, could have become mature enough to become insensitive to such a shift in attention. This would explain why we observed no effect of pubertal status for this task. In contrast, a more taxing task would be one that asks people to interpret higher order emotions, such as the RMET. Here one would expect that less efficient face processing would directly lead to worse performances for this RMET task. More research into this possible change in attentive processes around the onset of puberty is needed to come up with more specific hypotheses on how this change might influence social processes.

Another possibility is that the decline in performance is unrelated to pubertal status over all, and is instead due to changes in visual information processing not influenced by puberty onset. The dip in performance might for instance occur when the knowledge about faces is reorganized once a certain level of proficiency is reached (Flin, 1985). Transition from one phase to the other results in a temporary disruption in performance. Another developmental change in visual information processing is the change in sensitivity to details (for a review, see Van Den Boomen et al., 2012). Over development adolescents change from featural-oriented to configural-oriented face processing (Mondloch et al., 2002). This transition takes place as the ability to process low spatial frequency (LSF) increases with age. The use of LSF information during face processing results in better face perception and face recognition (Peters and Kemner, 2017). This switch in face processing might also explain our finding why we observe pubertal status effects in the RMET task, as emotion recognition is more reliant on LSF information (Vlamings et al., 2009). Maybe LSF processing was not yet fully developed in our prepubertal group, whereas it was in our midpubertal and postpubertal groups, which could explain the worse performance of the first group on the RMET. Gaze cueing on the other hand might rely less on LSF processing (Munsters et al., 2016), such that the switch in processing does not influence performance on the gaze cueing task. Therefore, we also did not observe any pubertal status effects on this task.

It is interesting to note that whenever we observed an effect of pubertal status the performances do not appear linear nor show a dip, but are more plateau-like, with improvements in performances only from prepuberty to midpuberty for the RMET and from prepuberty to postpuberty for empathizing abilities. The gaze cueing effect is more robust and does not significantly change over adolescence, although a trend was observed. The finding of plateau-like performance is also present in the face recognition literature. Various studies found a dip in performance, yet a leveling of performance was reported several times as well (for a review, see Chung and Thomson, 1995). These different findings may be due to methodological differences in the task paradigm. It therefore seems that the developmental curve for social processes indeed shows irregularities, yet inconsistency in results questions the reliability of the pubertal dip.

Further, we showed that sex differences in social behavior are not strongly present in our large sample of adolescents. Only in empathizing abilities did we observe sex differences, with girls showing higher scores than boys, but not in the gaze cueing task and the RMET. Apparently, in our results the sex difference in empathy does not extend to other social abilities such as gaze following and reading emotions from the eyes. The lack of a sex effect is in contrast with previous studies with adults which examined gaze following (Bayliss et al., 2005; Deaner et al., 2007) and emotion recognition (Alwall et al., 2010; Hall et al., 2010; Hoffmann et al., 2010; Kirkland et al., 2013; Baron-Cohen et al., 2015). An explanation may be that social and attentive processes are still under development across adolescence and only after the maturation of these processes the sex differences become prevalent in performances. However, sex differences in basic emotion recognition are previously reported in children (Lawrence et al., 2015). Clearly, these differences are inconsistent, with studies often requiring large sample sizes to find small effects. Therefore, a task that considers a wide range of complex emotions, such as the RMET, might not detect overall sex differences at this young age. In addition, adult studies into sex differences in the social domain show inconsistencies as well (for an overview, see Helgeson, 2017). Probably, a more nuanced view is needed where females excel in certain social skills whereas males excel in others, especially when aggressive stimuli are involved (for a review, see Forni-Santos and Osório, 2015). For other social skills a sex difference simply appears to be absent. More longitudinal studies into social processes are needed to unravel the developmental time course of possible sex differences.

To conclude, social behavior undergoes several changes over adolescence. This study shows improvements in emotion recognition from the eyes and empathizing abilities over pubertal development, although plateau-like. Gaze following on the other hand seems to be less influenced by pubertal status. Moreover, although girls outperformed boys on empathy abilities, sex differences were not prevalent in gaze following and emotion recognition from the eyes. Thus, we reveal developmental changes in these three abilities on social information processing across puberty, yet these do not pattern consistently across the different skills. As such, it is unlikely that gonadal hormones are exerting a simple and unified influence on all these abilities but rather that, if they do play a role in the development of these skills, the picture is more complex. Further research should therefore explore changes in (visual) information processing around puberty onset to find a more fitted explanation for changes in social behavior over adolescence.

## Author contributions

CK and CJ designed the experiments. RvR analyzed the data. RvR, CJ, and CK wrote the manuscript.

### Conflict of interest statement

The authors declare that the research was conducted in the absence of any commercial or financial relationships that could be construed as a potential conflict of interest.

## Footnotes

^1^We reclassified the children into three groups to be able to compare our results with those of Vetter et al. (2013). We also performed the analyses without this reclassification. This led to very similar results.
